# Comparison of Thermal and Electrical Modalities in the Assessment of Temporal Summation of Pain and Conditioned Pain Modulation

**DOI:** 10.3389/fpain.2021.659563

**Published:** 2021-09-27

**Authors:** Monica Sean, Alexia Coulombe-Lévêque, Martine Bordeleau, Matthieu Vincenot, Louis Gendron, Serge Marchand, Guillaume Léonard

**Affiliations:** ^1^Research Centre on Aging, School of Rehabilitation, Faculty of Medicine and Health Sciences, Université de Sherbrooke, Sherbrooke, QC, Canada; ^2^Department of Pharmacology-Physiology, Faculty of Medicine and Health Sciences, Université de Sherbrooke, Sherbrooke, QC, Canada; ^3^Department of Neurosurgery, Faculty of Medicine and Health Sciences, Université de Sherbrooke, Sherbrooke, QC, Canada

**Keywords:** pain, transcutaneous electrical nerve stimulation, thermode, cold pressor test, conditioned pain modulation, temporal summation of pain, dynamic quantitative sensory testing

## Abstract

Temporal summation of pain (TSP) and conditioned pain modulation (CPM) can be measured using a thermode and a cold pressor test (CPT). Unfortunately, these tools are complex, expensive, and are ill-suited for routine clinical assessments. Building on the results from an exploratory study that attempted to use transcutaneous electrical nerve stimulation (TENS) to measure CPM and TSP, the present study assesses whether a “new” TENS protocol can be used instead of the thermode and CPT to measure CPM and TSP. The objective of this study was to compare the thermode/CPT protocol with the new TENS protocol, by (1) measuring the association between the TSP evoked by the two protocols; (2) measuring the association between the CPM evoked by the two protocols; and by (3) assessing whether the two protocols successfully trigger TSP and CPM in a similar number of participants. We assessed TSP and CPM in 50 healthy participants, using our new TENS protocol and a thermode/CPT protocol (repeated measures and randomized order). In the TENS protocol, both the test stimulus (TS) and the conditioning stimulus (CS) were delivered using TENS; in the thermode/CPT protocol, the TS was delivered using a thermode and the CS consisted of a CPT. There was no association between the response evoked by the two protocols, neither for TSP nor for CPM. The number of participants showing TSP [49 with TENS and 29 with thermode (*p* < 0.001)] and CPM [16 with TENS and 30 with thermode (*p* = 0.01)] was different in both protocols. Our results suggest that response to one modality does not predict response to the other; as such, TENS cannot be used instead of a thermode/CPT protocol to assess TSP and CPM without significantly affecting the results. Moreover, while at first glance it appears that TENS is more effective than the thermode/CPT protocol to induce TSP, but less so to induce CPM, these results should be interpreted carefully. Indeed, TSP and CPM response appear to be modality-dependent as opposed to an absolute phenomenon, and the two protocols may tap into entirely different mechanisms, especially in the case of TSP.

## Introduction

Chronic pain affects approximately one-quarter of Canadians and remains a challenging condition for healthcare professionals (1). Several chronic pain conditions are characterized by alteration of endogenous pain modulation mechanisms, such as increased temporal summation of pain (TSP) and/or decreased conditioned pain modulation (CPM), which reflect excitatory and inhibitory mechanisms, respectively (2–6). TSP is characterized by an increase in pain intensity throughout a tonic or repetitive noxious stimulation of fixed intensity (7–9), while CPM is characterized by a widespread hypoalgesia following a noxious stimulation (10, 11). According to Yarnitsky (12), imbalances in TSP and CPM could predict response to certain treatments (13–15). Therefore, the assessment of TSP and CPM could help healthcare providers to personalize the treatment plan for patients with chronic pain.

Different protocols can be used to assess TSP and CPM using various types of modalities (thermal, mechanical, electrical, etc.) (16–19). One such protocol, which has been developed by our team (20), allows for the measurement of both TSP and CPM, by administering a test stimulus (TS) before and after a conditioning stimulus (CS): TSP is assessed by measuring the fluctuations in pain scores throughout the first instance of the TS, and CPM is assessed by calculating the difference in pain levels evoked by the TS before and after the CS. This protocol originally used thermal stimulation, with the TS consisting of a moderately painful tonic heat stimulation delivered for 120 s using a thermode, and the CS consisting of a cold pressor test (CPT), wherein the subjects immerse their dominant forearm in a cold-water bath (10°C) for 120 s. Unfortunately, this protocol, like most other TSP and CPM protocols, requires complex, costly, and time-consuming apparatus and procedures; as such, it is not a realistic option for routine clinical assessment (21).

To mitigate the need for a clinic-friendly TSP/CPM assessment protocol, our team has recently conducted an exploratory study wherein we used transcutaneous electrical nerve stimulation (TENS) as a substitute for the thermode and CPT used in the aforementioned protocol in healthy participants (22). TENS was chosen because it is simpler, more affordable, and easier to operate than a thermode and CPT. As such, that exploratory study aimed to compare the TSP and CPM responses obtained with both approaches (TENS vs. thermode/CPT). Results from that study suggested that both protocols were equivalent for the evaluation of TSP (though the response to one modality did not predict response to the other), but that the TENS protocol was less suited to induce CPM compared to the thermode/CPT protocol, most likely due to methodological issues (e.g., electrode location and contact area). These methodological issues have been addressed in the present study to make TENS a better candidate for the evaluation of TSP and CPM in a healthy population, which could pave the way for TSP and CPM to be routinely evaluated in the clinical setting, thereby facilitating clinical decision making (e.g., choice of pharmacological treatments) (12).

The objective of this study was to compare this new TENS protocol with the thermode/CPT protocol. More specifically, we aimed (1) to evaluate the association between the TSP magnitude obtained with the two protocols, (2) to evaluate the association between the CPM magnitude obtained with the two protocols, and (3) to assess whether the two protocols trigger significant TSP and CPM (10% change in pain levels) in a similar number of participants.

## Methods

This randomized cross-over study was conducted in accordance with the Declaration of Helsinki. All participants provided written informed consent for their participation. Ethics approval was granted from the institutional review board of the Centre intégré universitaire de santé et de services sociaux de l'Estrie–Centre hospitalier universitaire de Sherbrooke (CIUSSS de l'Estrie–CHUS), Sherbrooke, Canada (file number: 2019-3022). The trial has been registered at Clinicaltrial.gov (*NCT04236570*).

### Participants

Participants were eligible to take part in this study if they were free from pain before undergoing the sessions and between 18 and 59 years of age. Participants were excluded if they suffered from acute or chronic pain, depression, Raynaud's syndrome, neurological disorders, or musculoskeletal disorders (including forearm, knee, or ankle injuries); if they had previous experience with TENS; or if they presented contraindication to TENS (history of epilepsy, presence of a pacemaker, or metal implants) (23). Participants using antidepressants, anticonvulsants, or psychostimulants were also excluded.

Participants were asked to refrain from taking analgesics during the 7 days preceding each experimental session and to refrain from consuming caffeine and from doing intense physical activity during the 24 h preceding each experimental session.

### Sample Size

We determined that we needed a sample size of 50 to detect a correlation ≥0.40 (weak to medium association) between CPM/TSP magnitudes obtained with the two protocols, using a two-tailed test with a confidence level α set at 0.05.

### Study Design

Participants attended two experimental sessions at the Research Centre on Aging of the CIUSSS de l'Estrie-CHUS, during which the evaluation of TSP and CPM took place. In one session, TSP and CPM were evaluated using the new TENS protocol, while in the other one, TSP and CPM were evaluated using the thermode/CPT protocol. Session order was randomized between participants (randomization by blocks of four stratified by sex using a number table). The two experimental sessions were separated by 24 to 72 h, to minimize environmental/historical/hormonal differences between the two sessions (e.g., phase of the menstrual cycle) (24) while allowing sufficient time for CPM washout (25, 26). A single experimenter (female, MS) conducted all sessions.

### Experimental Procedure

**Figure 1:**
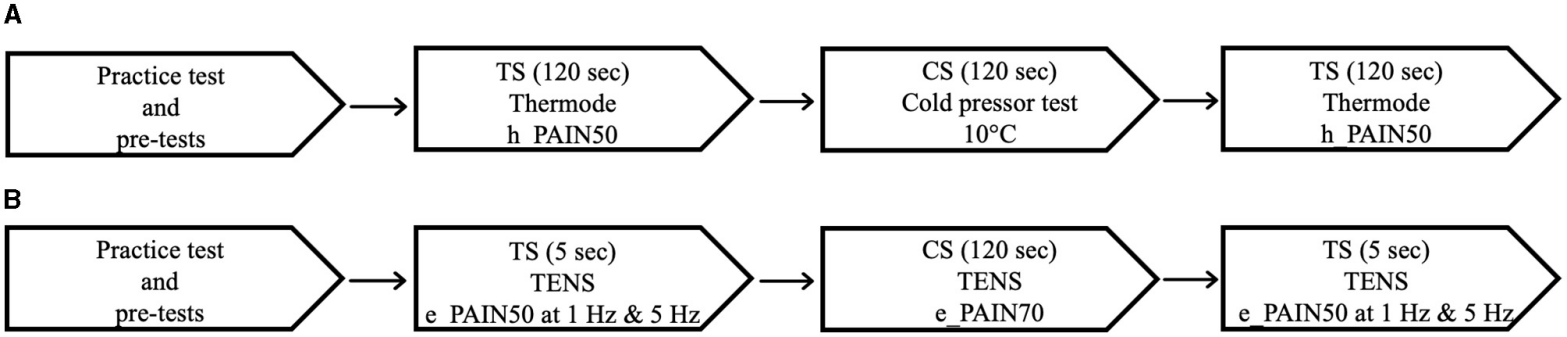
Testing sequence of the thermode/CPT protocol **(A)** and the TENS protocol **(B)**. CPT, cold pressor test; TENS, transcutaneous electrical nerve stimulation; CS, conditioning stimulus; TS, test stimulus.

An overview of the experimental procedure for both protocols is outlined in Figure 1. The first step of each protocol consisted of practice tests and pretests, which served to familiarize the participants with the apparatus and to establish individually-tailored stimulation intensities for the TS and CS. The testing procedure was then conducted; the TS, a moderately painful stimulation, was applied before and after the CS, a more painful stimulation serving to activate CPM. In the thermode/CPT protocol, the TS consisted of a heat stimulation applied with a thermode, and the CS consisted of a CPT (a cold-water bath in which the participants immersed their forearm). In the TENS protocol, both the TS and the CS consisted of electrical stimulation applied with TENS. TSP was measured as the increase in pain levels throughout the pre-CS TS, and CPM was measured as the difference in pain levels between the pre- and post-CS TS. In the following sections, for the two protocols, each step of the experimental procedure will be described in detail.

### Apparatus

#### Thermode/CPT Protocol

The TS was generated by a 3 cm^2^ thermode (TSA II, NeuroSensory Analyzer, Medoc Instruments, North Carolina, USA) applied for 120 s on the non-dominant forearm of each participant. Pain perception was continuously recorded with a computerized visual analog scale (CoVAS), which consists of a slider running along a 100 mm horizontal track housed in a box. Participants were asked to rate their pain by moving the slider between the left boundary (identified as “no pain” - score = 0) and the right boundary (identified as “worst pain imaginable” - score = 100). The CoVAS sampling rate was set at 10 Hz (10 pain measurements per second). The CS consisted of CPT (a cold-water bath in which the participants immersed their forearm and hand for 120 s) built by the in-house engineers to be at 10°C.

#### TENS Protocol

The TS and CS were generated by a TENS Eclipse Plus digital (Empi, St-Paul, Minnesota, USA) with carbon electrodes (4 cm^2^) instead of a thermode and CPT. The carbon electrodes were placed on the femoral condyles of each knee and over the two malleoli of the dominant ankle; the TS was applied on the non-dominant knee, and the CS on the dominant knee and the dominant ankle. The femoral condyles were our chosen electrode location as they are bony surfaces not adjacent to any major motor or sensory nerve; as such, TENS is unlikely to induce unpleasant or distracting muscle contractions, and habituation is less likely to take place (22, 27, 28). The TENS parameters were set to low-frequency, high-intensity mode (1 or 5 Hz, 400 μs for the TS; and 2 Hz, 400 μs for the CS). The TS consisted of two 5-s pulsed stimulations (at 1 and 5 Hz, respectively), and the CS consisted of a continuous, 120-s stimulation (2 Hz). Pain was assessed with a visual analog scale (VAS) of 100 mm, with the left boundary identified as “no pain” (score = 0) and the right boundary identified as “worst pain imaginable” (score = 100). The VAS was presented on a sheet of paper and the participants were asked to rate their pain intensity by tracing a line on the 100 mm line. The VAS was preferred to the CoVAS because it is much cheaper and better suited to the daily reality of clinical practice. Pain measurements were taken at 15, 30, 60, 90, and 120 s, always on a new sheet of paper.

Three main aspects of this TENS procedure differ from the exploratory study (22). First, the TS was applied on the non-dominant knee, as opposed to the ankle used in the exploratory study; this was done to avoid stimulating directly over a nerve (sural nerve), which could lead to habituation (28). Second, the CS was applied on the entire lower leg, as opposed to the ankle only in the exploratory study; this was to facilitate a larger CPM response (29). Third, the TS consisted of two short stimulations of 5 s each applied at different frequencies (at 1 and 5 Hz, respectively), instead of the continuous, 120-s stimulation at 2 Hz used in the exploratory study. This was because the continuous stimulation in the exploratory study had failed to elicit TSP (measured as the increase in pain levels throughout the stimulation) in a significant number of participants in the exploratory study, and the literature suggests that pulsed electrical stimulations can be used to measure TSP, by evaluating the difference in pain levels evoked by two stimulations applied at different frequencies (18).

### Practice Tests and Pretests

#### Thermode/CPT Protocol

The practice test allowed participants to become familiar with the apparatus and stimulation, and the pretests were used to identify the stimulation parameters to be used as the formal TS. For the practice tests and pretests, the thermode was applied on the anterior forearm of the non-dominant arm (the exact location was slightly varied between the tests to avoid habituation or sensitization), and the temperature was gradually increased from a baseline temperature (32°C) to a maximum of 51°C at a rate of 0.3°C/s. During the practice test, the participants were asked to verbally identify the point at which the heat became painful (heat pain threshold) and the point at which the pain was no longer tolerable (heat pain tolerance threshold). The purpose of the practice test was to familiarize participants with the apparatus and heat stimulation. During the pretests, pain perception was continuously recorded with the COVAS: participants were advised that the slider should remain at the left boundary while they felt no pain, that they should start moving the slider toward the right along the 100 mm horizontal track as the heat became painful (pain threshold), and that they should continue to move the slider to rate their pain until it was no longer tolerable (pain tolerance), at which point the slider should have reached the right boundary. The pretests served to identify the h_PAIN50 temperature, i.e., the temperature inducing pain levels of 50/100. For each participant, pretests were repeated until the h_PAIN50 temperature was consistent (within 1°C) between trials; the h_PAIN50 temperatures obtained from these trials were averaged and the resulting temperature was used for the formal TS.

#### TENS Protocol

The practice tests and pretests for the TENS protocol served the same purpose as for the thermode/CPT protocol: that is, the practice tests allowed participants to become familiar with the apparatus and stimulation, and the pretests were used to identify the stimulation parameters to be used for the TS, and, in the case of the TENS protocol, for the CS as well. Electrodes were placed on the femoral condyles of both the knees; on the non-dominant knee, the stimulation frequency was set at 1 Hz, and on the dominant knee at 2 Hz. For the practice test, the current was gradually increased from 0 mA to a maximum of 60 mA at a rate of 1 mA/s; the participants were asked to verbally identify the moment when the stimulations became painful (electrical pain threshold) and when it was no longer tolerable (electrical pain tolerance threshold). For the pretests, the stimulation was increased from 0 to the pain tolerance threshold at a rate of 1 mA/s, and participants were asked to rate their pain every second on a paper VAS (new VAS for each rating). The pretests were conducted separately on each knee; for the non-dominant knee, the aim was to identify the e_PAIN50 stimulation intensity, and for the dominant knee, the aim was to identify the e_PAIN70 stimulation intensity (i.e., the stimulation intensity inducing pain levels of 50/100 and 70/100, respectively). Pretests were repeated until the e_PAIN50 and e_PAIN70 of the participants were consistent (within 2 mA or less) between the trials. For each participant, the e_PAIN50 obtained from these trials were averaged and the resulting stimulation intensity was used for the formal TS of the participant; the e_PAIN70 simulation intensities were similarly averaged to yield the stimulation intensity to be used for the formal CS.

### Test Stimulus

#### Thermode/CPT Protocol

The TS in the thermode/CPT protocol consisted of painful thermal stimulation, applied with a thermode on the non-dominant anterior forearm for 120 s at the predetermined, individually tailored temperature (h_PAIN50). Participants were told that the thermode temperature could increase, remain stable, or decrease throughout the stimulation (to avoid them forming specific expectations), and they were asked to continuously record their pain level using the CoVAS. In fact, after a constant rise (0.3°C/s) from baseline (32°C) to the predetermined temperature (h_PAIN50), the temperature remained constant throughout the entire TS (120 s). The TS was administered before the CS (pre-CS TS) and immediately afterwards (post-CS TS).

#### TENS Protocol

The TS in the TENS protocol consisted of a painful electrical stimulation applied with a TENS unit (with two electrodes placed on the femoral condyles of the non-dominant knee) at the predetermined, individually tailored e_PAIN50 current amplitude. Unlike the TS in the thermode/CPT protocol, which consisted of a constant stimulation applied for 120 s, the TS in this protocol consisted of two 5-s stimulations: the first at 1 Hz, and the second at 5 Hz (which allowed for the measurement of TSP, see *Measure of TSP*). Participants were asked to rate their level of pain after each 5-s stimulation by tracing a line on a paper VAS (new sheet of paper for each stimulation). As in the thermode/CPT protocol, this two-part TS was administered once before the CS (pre-CS TS) and again immediately afterwards (post-CS TS).

### Conditioning Stimulus

#### Thermode/CPT Protocol

The CS in this protocol consisted of a CPT, wherein the participants immersed their dominant forearm in a cold-water bath at 10°C for 120 s. During the CPT, participants verbally rated the intensity and unpleasantness of their pain on a 100-point numerical pain scale (with the same anchors as the CoVAS), at *t* =15, 30, 60, 90, and 120 s. These scores were used to calculate the average pain evoked by the CS.

#### TENS Protocol

The CS in the TENS protocol consisted of a noxious electrical stimulus generated by a TENS unit, with four electrodes placed on the femoral condyles and ankle malleoli of the dominant leg. The stimulation was first applied to the two knee electrodes at the predetermined, individually-tailored e_PAIN70 current amplitude; the stimulation was then applied to the two ankle electrodes, with the stimulation intensity gradually increasing up until the participants rated the pain evoked by the ankle stimulation as matching the intensity of the pain evoked by the knee stimulation. The stimulation was then maintained over the four electrodes for 120 s. As in the thermode/CPT protocol, the participants verbally rated the intensity and unpleasantness of their pain on a 100-point numerical pain scale (with the same anchors as the VAS), at *t* =15, 30, 60, 90, and 120 s during the CS. These scores were used to calculate the average pain evoked by the CS.

### Measure of TSP

#### Thermode/CPT Protocol

In this protocol, TSP was measured by evaluating pain fluctuations during the pre-CS TS. More specifically, TSP was obtained by tracing a linear regression obtained from the pain scores at *t* = 30, 60, 90, and 120 s (22). The slope of that linear regression was used as a magnitude of TSP, such that positive scores represented increased activation of TSP.

#### TENS Protocol

TSP was measured by evaluating the difference in pain levels evoked by the two 5-s pre-CS TS stimulations, which were applied at different frequencies (see *Test-Stimulus*). More specifically, the magnitude of TSP (delta pain scores) was calculated by subtracting the 1 Hz pre-CS TS pain score from the 5 Hz pre-CS TS pain score, such that a positive value indicated the presence of TSP. This method of obtaining TSP scores differs significantly from the method used to measure TSP in the exploratory study (22), which measured changes in pain levels throughout a continuous, 120 s electrical stimulation. This new method was chosen based on results obtained by Marouf et al. (18).

### Measure of CPM

#### Thermode/CPT Protocol

In the thermode/CPT protocol, the pain intensity ratings obtained throughout each 120-s TS (CoVAS sampling rate of 10 Hz) were averaged to yield a single pain intensity score for each TS (pre- and post-CS TS). The magnitude of CPM (delta pain scores) was measured by subtracting the average pre-CS TS pain score from the average post-CS TS pain score, such that a negative value indicated the presence of CPM.

#### TENS Protocol

The magnitude of CPM was measured by subtracting pre-CS TS pain score (calculated as the average between the 1 and 5 Hz pre-CS pain scores) from the post-CS TS pain score (calculated as the average between the 1 and 5 Hz post-CS pain scores), such that a negative value indicated the presence of CPM.

### Statistical Analysis

Normality was assessed using Shapiro-Wilk tests. Since normality could be assumed for all data distributions, parametric tests were used. Statistical significance was set at 0.05. Descriptive statistics are presented as mean ± SD. All analyses were conducted using SPSS Statistics (version 27).

The association between TSP magnitudes in the TENS protocol and TSP magnitudes in the thermode/CPT protocol was assessed with the Pearson coefficient (two-tailed). The same analysis was conducted for CPM.

The scores of TSP and CPM (continuous variable) were also transformed into a dichotomic variable, to determine whether one protocol was more effective than the other at evoking TSP or CPM. That is, for each participant, the TSP and CPM response to both protocols was classified as “present” or “absent” based on an objective threshold (22). For TSP in the TENS protocol (calculated as the delta in pain scores between the 5 Hz stimulation and the 1 Hz stimulation), this threshold was set at 10/100, such that a delta pain score larger than 10 percentage points was classified as “TSP present.” For TSP in the thermode/CPT protocol (calculated as the slope of the linear regression of the pain scores throughout the TS), this threshold was set at 0.1, such that a slope ≥0.1 (corresponding roughly to a pain increase of 10 percentage points) was classified as “TSP present.” For CPM (which in both protocols was calculated as the difference in pain levels evoked by the TS before and after the CS), the threshold was set at 10/100, such that a reduction in pain levels of 10 percentage points or more was classified as “CPM present.” These thresholds were chosen as changes in pain scores smaller than 10 percentage points are likely attributable to random fluctuations and not representative of an actual change in pain perception (22). McNemar's test was carried out to identify whether more participants showed TSP or CPM in one protocol compared to the other (sample-dependent). As an additional measure of association, the Phi coefficient (mean square contingency coefficient) was calculated to assess the consistency of the response to both protocols (i.e., whether the presence of CPM/TSP in one protocol was associated with the presence of CPM/TSP in the other protocol).

## Results

### Participants and Baseline Data

Fifty participants (biological sex: 25 men and 25 women; 48 Caucasians) aged 38 ± 12 years old took part in our study. The average thermode temperature used for the TS (h_PAIN50) was 46.7 ± 1.6°C, and the average TENS current (e_PAIN50) was 45 ± 12 mA. The average TENS current used for the CS (e_PAIN70) was 55 ± 8 mA at the knee and 51 ± 11 mA at the ankle.

**Figure 2:**
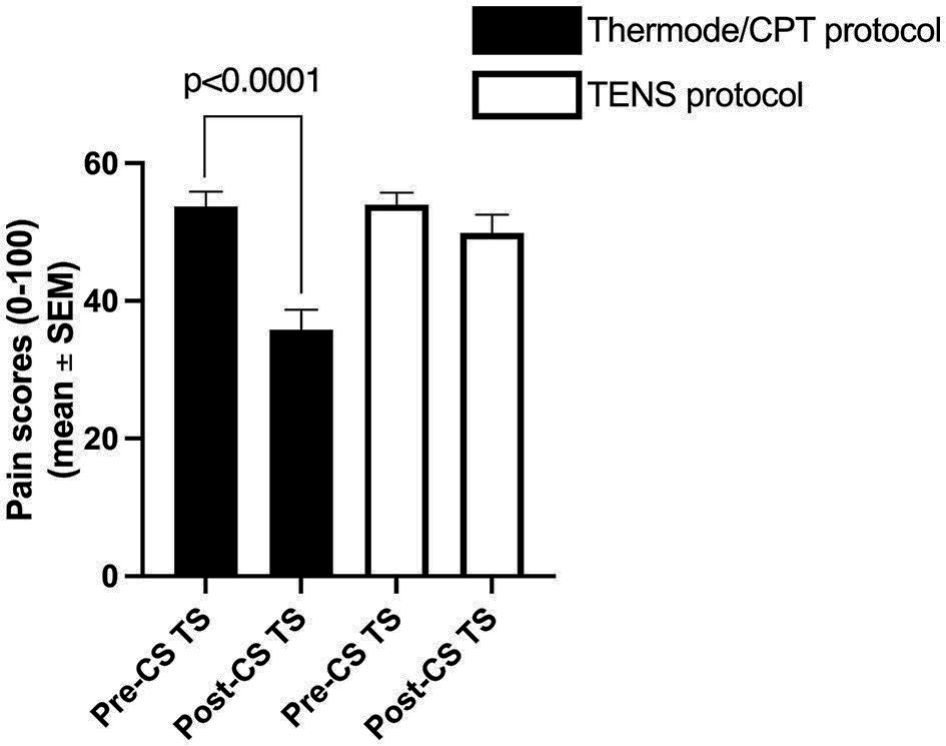
Average pain levels elicited by the thermode/CPT protocol and TENS protocol throughout the pre- and post-CS TS. CPT, cold pressor test; TENS, transcutaneous electrical nerve stimulation; CS, conditioning stimulus; TS, test stimulus.

**Figure 3:**
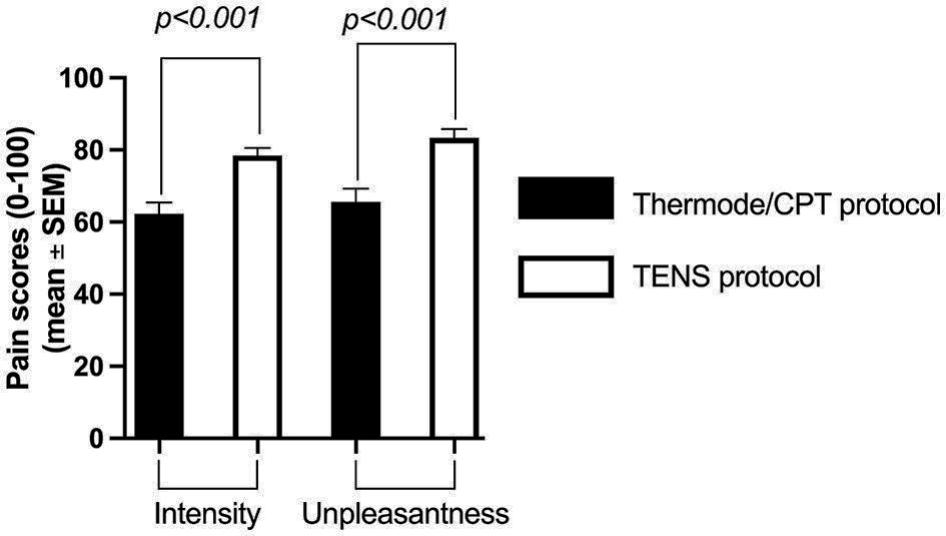
Average pain intensity and unpleasantness of pain induced by the CS with the thermode/CPT protocol and the TENS protocol. CPT, cold pressor test; TENS, transcutaneous electrical nerve stimulation.

The average pain intensity induced by the pre-CS TS was similar in both protocols (54 ± 15/100 with the thermode/CPT protocol and 54 ± 13/100 with the TENS protocol; *p* = 0.94; Figure 2). However, the average pain intensity induced by the CS was different between the two protocols (62 ± 22/100 with the CPT and 78 ± 15/100 with TENS; *p* < 0.001), as was the average unpleasantness of pain evoked by the CS (66 ± 26 /100 with the CPT and 83 ± 16/100 with the TENS; *p* < 0.001; Figure 3).

### Temporal Summation of Pain

**Figure 4:**
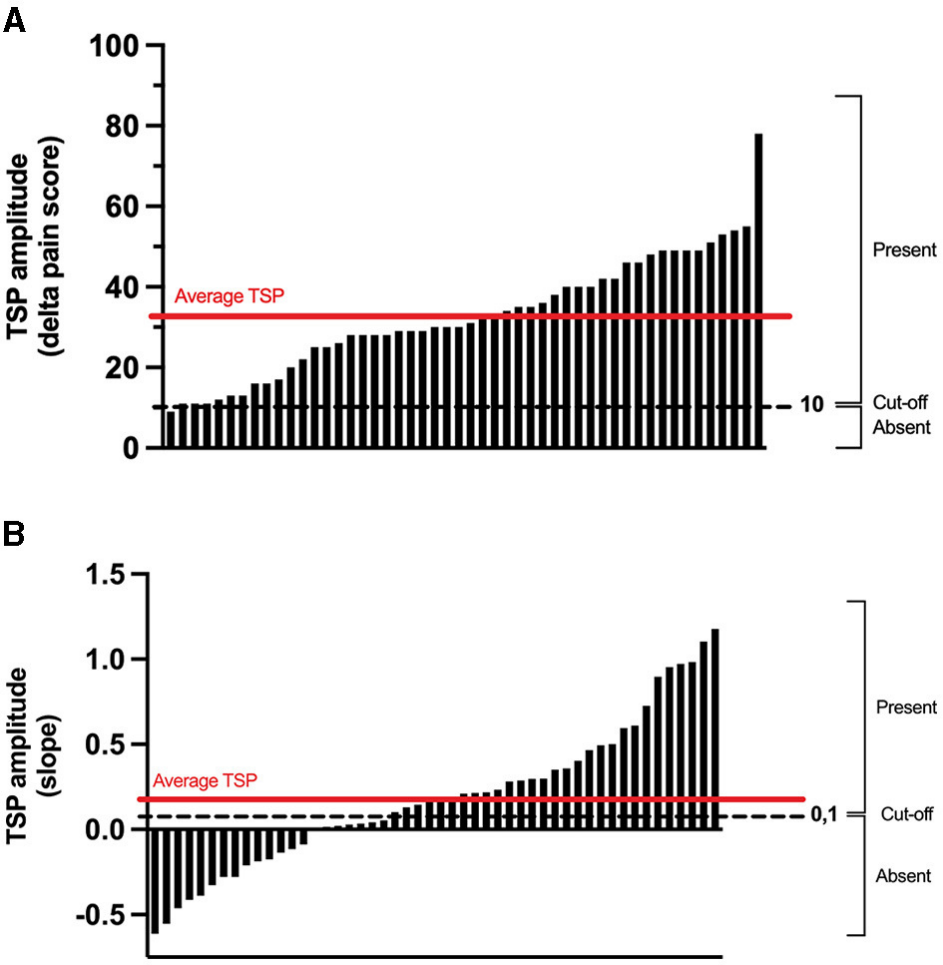
Individual TSP obtained during the pre-CS TS in the TENS protocol **(A)** and in the thermode/CPT protocol **(B)**. For TSP in the TENS protocol (calculated as the delta in pain scores between 5 Hz stimulation and 1 Hz stimulation), a delta pain score larger than 10 percentage points was classified as “TSP present.” As for the TSP in the thermode/CPT protocol (calculated as the slope of the linear regression of the pain scores throughout the TS), a slope larger than 0.1 was classified as “TSP present.” CPT, cold pressor test; TENS, transcutaneous electrical nerve stimulation; CS, conditioning stimulus; TS, test stimulus; TSP, temporal summation of pain.

The average TSP magnitude was 33 ± 15/100 with the TENS protocol (delta pain score) and 0.19 ± 0.43 with the thermode/CPT protocol (linear regression slope) (Figure 4). There was no correlation (*r* = 0.11; *p* = 0.45) between TSP magnitudes induced by the two protocols. There was also no association between the presence of TSP in the TENS protocol and the presence of TSP in the thermode/CPT protocol (Φ = 0.17; *p* = 0.24), with 30 out of 50 (i.e., 60%) participants having the same response to both protocols (TSP present in both protocols or TSP absent in both protocols; refer to Table 1). The two protocols evoked TSP in a different number of participants: 49 participants out of 50 (98%) had TSP in the TENS protocol, compared to only 29 (58%) in the thermode/CPT protocol (*p* < 0.001).

**Table 1:** TSP and CPM scores (continuous variables) were transformed into “present/absent” scores (dichotomic variables).

		**TSP**	**CPM**
Same response to both protocols	Present with both protocols	29	9
			
	Absent with both protocols	1	13
			
Different response to the two protocols	Present with only thermode	0	21
			
	Present only with TENS	20	7
			
Statistical analysis	McNemar's *p*	*P* < 0.001	*p* = 0.01
	Phi coefficient	Φ = 0.17; *p* = 0.24	Φ = −0.053; *p* = 0.71

*The consistency of response (i.e., whether participants had the same “present/absent” score with both protocols) is reported for TSP (left column) and CPM (right column). For both TSP and CPM, the Phi coefficient (mean square contingency coefficient) was calculated to assess the association between response to the TENS protocol and response to the thermode/CPT protocol. TSP, temporal summation of pain; CPM, conditioned pain modulation; TENS, transcutaneous electrical nerve stimulation; CPT, cold pressor test*.

### Conditioned Pain Modulation

**Figure 5:**
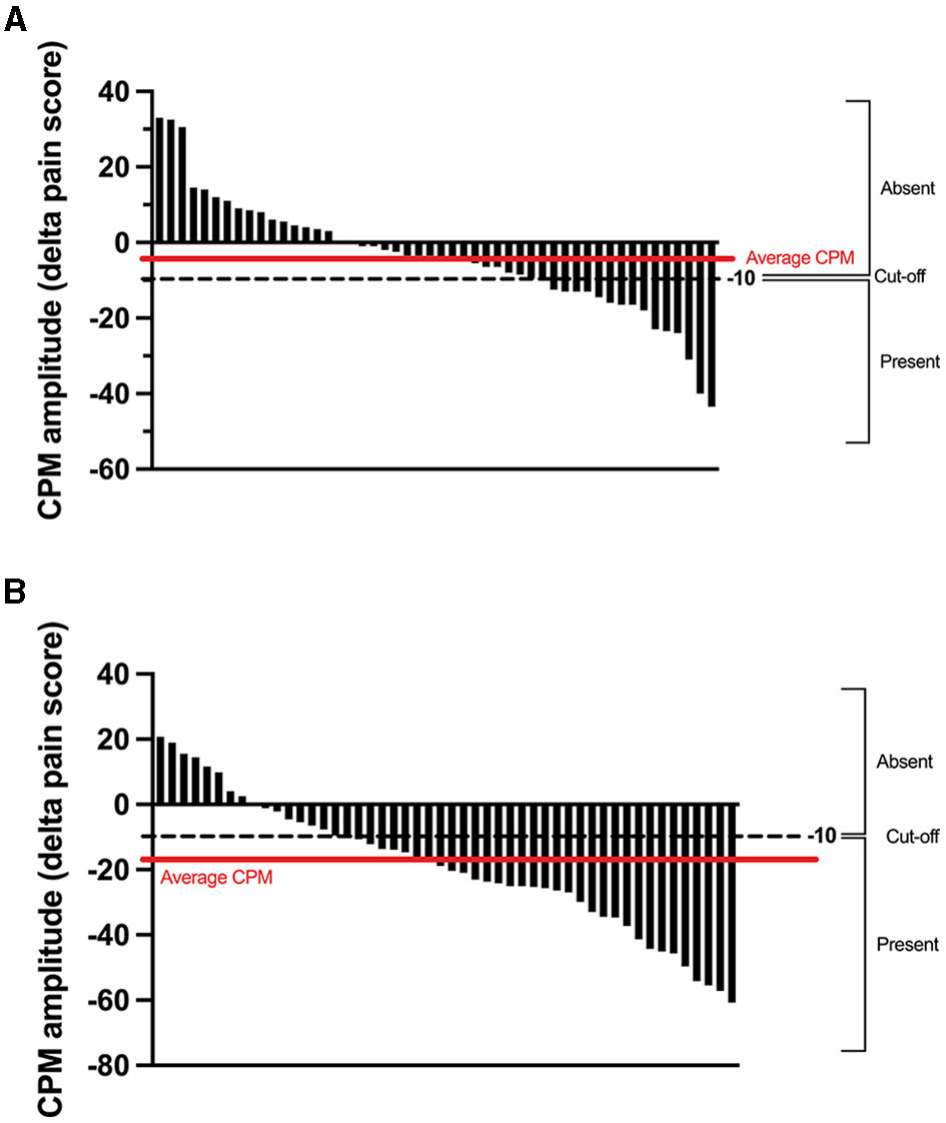
Individual CPM obtained by subtracting the pre-CS TS pain score from the post-CS TS pain score in the TENS protocol **(A)** and the thermode/CPT protocol **(B)**. For CPM in the TENS protocol and the thermode/CPT protocol (calculated as the difference in pain levels evoked by the TS before and after the CS), a reduction in pain levels of 10 percentage points or more was classified as “CPM present.” CPM, conditioned pain modulation; CPT, cold pressor test; TENS, transcutaneous electrical nerve stimulation; CS, conditioning stimulus; TS, test stimulus.

The average CPM magnitude (delta pain score) was −4 ± 16/100 in the TENS protocol and −18 ± 18/100 in the thermode/CPT protocol (Figures 2, 5). There was no correlation (*r* = 0.02; *p* = 0.89) between CPM magnitudes induced by the two protocols. There was also no association between the presence of CPM in the TENS protocol and the presence of CPM in the thermode/CPT protocol (Φ = −0.053; *p* = 0.71), with 22 out of 50 (i.e., 44%) participants having the same response to both protocols (CPM present in both protocols or CPM absent in both protocols; refer Table 1). CPM was evoked in 16 participants out of 50 (32%) in the TENS protocol, compared to 30 participants (60%) in the thermode/CPT protocol (*p* = 0.01).

## Discussion

The objective of this study was to compare the thermode/CPT protocol with the TENS protocol, by (1) measuring the association between the TSP evoked by the two protocols; (2) measuring the association between the CPM evoked by the two protocols; and (3) assessing whether the two protocols successfully trigger TSP and CPM in a similar number of participants. These objectives were reminiscent of those of our exploratory study (22), as we were hoping that the new, modified TENS protocol would be more successful than the original TENS protocol in inducing TSP/CPM. Indeed, a clinic-friendly method for the measurement of TSP/CPM would have a high clinical value, as it would provide physicians with valuable information to inform their decision-making (e.g., choice of medication). Unfortunately, despite these methodological modifications, results from the present study mostly replicate those of the exploratory study.

We found no association between the TSP evoked by the two protocols, be it in terms of the magnitude of TSP in both protocols (continuous variable), or in terms of the presence of TSP in both protocols (dichotomic variable). The same lack of association between the two protocols was found for CPM. These results, which replicate those of our exploratory study (22), suggest that one protocol cannot be used as a substitute for the other without a significant effect on the outcome. Our results also appear to suggest that the TENS protocol can trigger TSP in a larger number of participants than the thermode/CPT protocol, while the thermode/CPT protocol is more effective at triggering CPM. However, these results should be interpreted with caution.

The lack of association between the response to the TENS protocol and the response to the thermode/CPT protocol echoes the findings from our exploratory study (22) and other studies (30, 31). Altogether, these findings further support the interpretation that response to one protocol does not predict the response to the other protocol. The implication of this result, which has already been discussed in our exploratory study (22), is important enough that it bears repeating in this study. Indeed, a growing body of evidence suggests that chronic pain patients with “increased TSP” respond preferentially to certain classes of drugs, while others with “decreased CPM” respond preferentially to others (12–15). However, these studies tend to assess TSP and CPM in patients using a single modality. As we have seen, TSP and CPM do not seem to be “absolute” phenomena ready to be measured in any which way; on the contrary, TSP and CPM responses appear to be heavily affected by the type of modality used in their assessment. Further research is, therefore, required to evaluate to what extent TSP and CPM are found to be absolute phenomena, and to what extent they are modality-specific. In the meantime, clinicians should make sure that any TSP/CPM assessment they perform uses the same modalities as the predictive studies on which they are basing their decision-making.

Our results also suggest that the new TENS protocol remains less effective than the thermode/CPT protocol to evoke CPM. This is somewhat surprising, given the changes we made to the original TENS protocol (applying the CS on a larger area), which should have improved its performance. As such, these results could mean either that further tweaking of the TENS protocol is required to achieve a better CPM, or that TENS is simply not suited as a TS and/or CS to evoke CPM. There is some evidence in favor of both hypotheses.

In terms of stimulation parameters, CS location and CS intensity come to mind. Indeed, while the CS was applied over a larger area in the new TENS protocol compared to the original TENS protocol, in both cases, the CS was applied to the dominant leg; in contrast, in the thermode/CPT protocol, the CS was applied to the dominant arm and hand. Given that the density of sensory nerves is significantly higher in the arm and hand than in the leg (32), it is possible that TENS underperformed the CPT in evoking CPM simply because it was applied over a less richly innervated body area. Moreover, the pain intensity and unpleasantness evoked by the CS was greater in the TENS protocol than in the thermode/CPT protocol by roughly 15 percentage points. While one might instinctively expect that a more intense CS would induce a more potent CPM, this may not necessarily hold (33). Moreover, painful electrical stimulations activate the limbic system (notably the superior caudate nucleus and posterior insula) to a greater extent than painful thermal stimulations (34), suggesting that they have a stronger affective component than thermal stimuli (35). This could explain why participants found the TENS more unpleasant than the CPT, which in turn could have induced a state of anxiety or otherwise sensitized the central nervous system to the nociceptive stimuli, thereby counteracting CPM mechanisms.

We also have to consider the possibility that electrical stimuli in general, or TENS in particular, are simply less suited than other types of stimulation to evoke CPM. Indeed, as mentioned previously, different modalities (thermal, mechanical, electrical, etc.) used as TS and/or CS appear to vary in their ability to evoke CPM or TSP (30, 36–38). While these studies differ somewhat in the specifics of their findings, a general emerging trend suggests that electrical stimulation (either as the TS, the CS, or both) tends to be less suited than other types of stimulation to evoke CPM.

Our results also appear to suggest that the new TENS protocol is more effective than the thermode/CPT protocol to evoke TSP. However, once again, we urge the reader to use caution when interpreting these results, seeing as two elements relating to TSP differ between the two protocols: stimulation type and stimulation temporality. Indeed, the TENS protocol used an electrical stimulation as the TS, applied at two different frequencies to measure TSP; in contrast, the thermode/CPT protocol used a thermal stimulation as the TS, applied continuously for 120 s to measure TSP. As with CPM, it is possible that the difference in outcome between the two protocols is attributable to the type of modality (37, 39), possibly because electrically-induced and thermally-induced TSP appears to be mediated by different classes of sensory neurons (30, 40). However, given that our exploratory study (22) did not find that TENS was superior to the thermode to induce TSP, it seems more likely that it was not the difference in modality, but rather the difference in the “temporality” (varying frequencies vs. continuous) between the two TS that was responsible for the difference in TSP between the two protocols.

Our decision to evaluate TSP using two short stimulations of different frequencies, as opposed to prolonged and sustained stimulation, was based on recommendations from Marouf et al. (18). However, this change in the temporality of the TS might have been so extensive as to cause the two TS to tap into entirely different neurophysiological processes, and what we call “TSP” in response to the two TS could be two separate and independent mechanisms. Indeed, the TS delivered with the thermode is truly continuous, in that the firing rate of whichever first-order sensory neurons are activated is solely “set” by the said neurons. In contrast, the TS delivered by the TENS, which consists of an electrical impulse delivered at 1 Hz for 5 s at a set intensity, and subsequently at 5 Hz for 5 s at the same intensity, artificially constrains the firing rate of the first-order sensory neurons. As such, we cannot assume that the TSP evoked by these two types of stimulations arises from the same neurophysiological mechanism. In turn, while TENS appears to evoke “TSP” in a larger portion of participants than the thermode, if that “TSP” is a different phenomenon from the “TSP” evoked by the thermode, and from the “TSP” studied for its potential use in clinical decision-making, then by no means can the TENS be used to replace the thermode.

### Perspectives for Further Studies

This is our second study suggesting that TENS is less apt than the thermode/CPT protocol to assess CPM. However, we are not yet ready to throw in the proverbial towel; TENS may yet be able to evoke CPM, with the CS applied on a different location: the arm. Indeed, given that the arm and especially the hand is more richly innervated than the leg (32), a CS consisting of TENS applied on the arm and hand could conceivably be more effective at evoking CPM. As for TSP, we cannot be certain that the “TSP” obtained with the TENS is the same physiological “TSP” obtained with a thermode. We see two options to advance the research on this issue. First, we could revert to a 120 s electrical stimulation as was used in the exploratory study but set at a *higher frequency*, specifically, higher than the maximal impulse rate of the first-order sensory neurons, such that their firing rate is not externally constrained. A second approach would be to focus on the outcome rather than the physiological mechanism. If the TSP evoked by the pulsed TENS could be shown to have the same associations and predictive capabilities [e.g., predicting response to treatment (12)] as the TSP evoked by the thermode, then the issue as to whether both tap into the same mechanism would be moot; this second wave of research showing “TENS TSP” to behave similarly to “thermode TSP” would be enough to support using TENS to measure TSP in the clinic.

The present study did not include participants suffering from chronic pain as we were still trying to establish our protocol in a healthy population [TSP and CPM are known to differ between the healthy and chronic pain populations (41, 42)]. However, including a group of chronic pain participants would have allowed us to test our protocol in the population likely to benefit from it in the clinical setting [e.g., fibromyalgia (43) and chronic lower back pain (44)]. This would have allowed us to ensure that the protocol was well-tolerated in that population and that the protocols were equivalent in the healthy and patient population. Future studies should consider including patients with chronic pain in their sample.

Finally, no analyses were conducted regarding the effect of biopsychosocial characteristics (age, sex, BMI, etc.) on TSP and CPM, although these characteristics are known to affect TSP and CPM. These variables were not included because our focus was on whether the CPM/TSP response of each individual was the same in the two protocols. We were not trying to determine which participants had smaller/larger CPM/TSP, and we were also not attempting to identify the predictive factors correlating with the CPM/TSP response to one or both protocols. However, in “ignoring” the biopsychosocial characteristics, we were assuming that they would have a similar impact on the results from both protocols. However, this may not necessarily be the case; seeing as TSP/CPM appear to be modality-dependent, it is entirely possible that the effect of biopsychosocial characteristics on TSP/CPM is also modality-dependent. This would certainly be worth considering when designing any protocol to measure TSP/CPM.

## Conclusion

Our results, which replicate those of our exploratory study, suggest that response to one modality does not predict response to the other; as such, TENS cannot be used instead of a thermode/CPT protocol to assess TSP and CPM without significantly affecting the results. Moreover, while at first glance it appears that TENS is more effective than the thermode/CPT protocol to induce TSP, but less so to induce CPM, these results should be interpreted carefully. Indeed, TSP and CPM response appear to be modality-dependent as opposed to absolute phenomena; moreover, it is possible that the two protocols tap into entirely different mechanisms, especially in the case of TSP.

## Data Availability Statement

The raw data supporting the conclusions of this article will be made available by the authors, without undue reservation.

## Ethics Statement

The studies involving human participants were reviewed and approved by the institutional review board of the Centre intégré universitaire de santé et de services sociaux de l'Estrie–Centre hospitalier universitaire de Sherbrooke (CIUSSS de l'Estrie–CHUS), Sherbrooke, Canada. Participants provided their written informed consent to participate in this study.

## Author Contributions

MS and GL designed the study. MS carried out the experiment. All authors discussed the results and contributed to the final manuscript.

## Funding

This study was funded in part by the Chronic Pain Network, through the Canadian Institutes of Health Research (CIHR) funded Strategy for Patient-Oriented Research (SPOR) under the grant 358 108, as well as by the Centre de recherche du CHUS and the Faculty of Medicine and Health Sciences of the Université de Sherbrooke. GL and LG receive salary support by the Fonds de Recherche du Québec—Santé (FRQS).

## Conflict of Interest

The authors declare that the research was conducted in the absence of any commercial or financial relationships that could be construed as a potential conflict of interest.

## Publisher's Note

All claims expressed in this article are solely those of the authors and do not necessarily represent those of their affiliated organizations, or those of the publisher, the editors and the reviewers. Any product that may be evaluated in this article, or claim that may be made by its manufacturer, is not guaranteed or endorsed by the publisher.
